# Editorial for the Special Issue on High-Power Lasers for Materials Processing

**DOI:** 10.3390/mi14051041

**Published:** 2023-05-12

**Authors:** Patrice Salzenstein

**Affiliations:** Centre National de la Recherche Scientifique (CNRS), Franche-Comté Electronique Mécanique Thermique Optique Sciences et Technologies (FEMTO-ST) Institute, Université de Franche-Comté (UFC), F25030 Besançon, France; patrice.salzenstein@cnrs.fr

Power lasers have been around for a long time. Many lasers are available from different laser manufacturers. Lasers of high average power have multiple applications for working materials (drilling, sanding, hardening, welding, or cutting, etc.). The principle of laser welding is based on the fusion of a point of the material on which the beam will concentrate, thanks to the optical system. After focusing, its illumination can reach more than 1 MW/cm^2^. Lasers can be used for surface treatments. They can also be used to characterize the nature of materials by interacting with the medium, for example, to form phononic waves in the material and allow the material to respond. Accordingly, this Special Issue seeks to showcase research papers, as well as communications, that focus on the efforts made to solve problems, cut and treat surfaces, characterize materials, or any other application of these lasers.

Out of the seven articles published in this volume of this Special Issue, all of them are original research papers. Three papers were submitted from China, two were submitted from Germany, one was submitted from the United Kingdom, and one paper was contributed from Turkey.

In this Special Issue on miniature optoelectronic resonators and oscillators, we include seven papers, covering different aspects related to oscillating-amplifying integrated fiber lasers (1), calibration method for the resolution of two-dimensional laser direct writing (2), algorithms for weld depth measurement in laser welding of copper (3), highly integrated cladding mode stripper array (4), quasi-continuous wave pulsed laser welding of copper lap joints using spatial beam oscillation (5), in-process analysis of melt pool fluctuations with scanning optical coherence tomography for laser welding of copper (6), and a real-time working method that improves process efficiency in high-power fiber laser systems (7).

In particular, Donglin Yan et al. describe A 3.7-kW oscillating-amplifying integrated fiber laser, featuring a compact oval-shaped cylinder package [1]. Xu Yie et al. present a calibration method for the resolution of two-dimensional TPP laser direct writing [2]. Thomas Will et al. report algorithms for weld depth measurement in laser welding of copper with scanning optical coherence tomography [3]. Yu Liu et al. demonstrate a highly integrated cladding mode stripper array for compact high-power industrial fiber laser [4]. The research article by Amirhossein Sadeghian et al., in this issue, highlights quasi-continuous wave pulsed laser welding of copper lap joints using spatial beam oscillation [5]. In a second article of this issue, Thomas Will et al. report in-process analysis of melt pool fluctuations with scanning optical coherence tomography for laser welding of copper for quality monitoring [6]. Additionally, Uğur Yalçın and Uğur Karanfil feature the development of a real-time working method that improves process efficiency in high-power fiber laser systems [7].

We hope that this Special Issue for Micromachines will offer readers a good overview of the current state-of-the-art in this fast-growing area of research, as well as an introduction to some of the newest techniques developed in the field.
